# A DFT Study on Be_n_(n = 10–12) Clusters with Hydrogen Storage Capacity

**DOI:** 10.3390/molecules31030566

**Published:** 2026-02-06

**Authors:** Chunyu Yao, Shunping Shi, Zhanjiang Duan, Xiaoling Liu, Kai Diao, Jiabao Hu, Deliang Chen

**Affiliations:** 1 Department of Physics, College of Physics, Chengdu University of Technology, Chengdu 610059, China; 13568172976@163.com (C.Y.); dzj278632@163.com (Z.D.); 18228879359@163.com (X.L.); pengpeigen@861china.com (J.H.); 2 Yibin Campus, Chengdu Technological University, Yibin 644000, China; dkai1@cdtu.edu.cn; 3 School of Physics and Electronic, Guizhou Education University, Guiyang 550018, China; chendeliang@gznc.edu.cn

**Keywords:** hydrogen storage, Be_n_(n = 10–12), hollow spheres, density functional theory

## Abstract

Hydrogen energy has garnered widespread attention as a clean energy source. This study employs density functional theory (DFT) to systematically investigate the hydrogen storage performance of Be_n_(n = 10–12) clusters. The results reveal that hollow spherical Be_n_ clusters exhibit excellent hydrogen storage capacity while maintaining good thermal stability even after H_2_ adsorption at room temperature. Specifically, Be_10_, Be_11_ and Be_12_ clusters can adsorb 26, 28, and 30 H_2_ molecules, achieving hydrogen storage densities of 31.96 wt%, 31.87 wt%, and 35.87 wt%, respectively—far exceeding the U.S. Department of Energy’s target of 5.5 wt%. Calculations indicate an average adsorption energy between 0.16 and 0.19 eV/H_2_, which lies between physisorption and chemisorption. IGMH isosurface analysis confirms the physisorption characteristics of H_2_ molecules. PDOS analysis reveals that the hydrogen storage mechanism primarily originates from H_2_ molecular polarization and van der Waals forces arising from orbital hybridization between hydrogen atoms and the substrate. Desorption temperature calculations show that, above 216 K, this material demonstrates potential for reversible hydrogen storage. This study demonstrates that these three hollow spherical beryllium cluster systems are ideal candidates for achieving ultra-high-capacity reversible hydrogen storage.

## 1. Introduction

As the global population grows and living standards improve, the finite nature of traditional energy sources and their overuse have led to energy shortages and environmental pollution. Renewable energy sources are considered promising long-term solutions to meet future energy demands [1,2,3]. Among these, hydrogen and solar energy are primary renewable options. Given the uneven distribution of solar energy, hydrogen energy stands out as the most suitable long-term energy supplier due to its highest volumetric energy density. Hydrogen is the most abundant element in the universe and serves as a secondary energy source with numerous advantages, including high combustion heat, abundant availability, and environmental friendliness [4]. Among renewable fuels, hydrogen is one of the most preferred future fuels because it produces zero emissions when burned with atmospheric oxygen. Additionally, hydrogen’s energy density (142 MJ/kg) is three times that of gasoline (46 MJ/kg), and its combustion product is water [5]. Due to its abundance, versatility, and zero greenhouse gas emissions during combustion, hydrogen is a promising and environmentally friendly energy source for the future. Hydrogen storage technology is crucial for advancing the hydrogen fuel economy [6]. However, the challenge of efficient and safe hydrogen storage remains a significant barrier to widespread adoption. Traditional storage methods, such as compressed gas and liquid hydrogen, face limitations in energy density, safety, and cost, necessitating the search for alternative hydrogen storage materials with superior weight and volume capacities [7,8].

Hydrogen storage materials play a vital role in the development of a hydrogen fuel economy. However, achieving a perfect balance between safety, storage capacity under ambient conditions, and cost-effective production remains a major challenge that must be addressed before commercialization [9,10,11,12,13]. Solid-state hydrogen storage technology involves the physical or chemical binding of hydrogen with storage materials to achieve hydrogen storage. Considering factors such as volumetric hydrogen storage density and safety, solid-state hydrogen storage is one of the most commercially promising storage methods. This includes various types such as metal hydrides, complex hydrides, metal–organic frameworks (MOFs), and carbon-based materials [14]. Metal hydrides, as a mainstream technology in solid-state hydrogen storage, involve materials such as magnesium alloys, vanadium alloys, and rare earth alloys [15]. Among metal hydrides, most researchers focus on lightweight elements to develop new hydrogen storage materials [5,16,17,18]. Magnesium alloys, for instance, offer high hydrogen storage capacities (up to 7.6 wt%) [19,20]. Gupta et al. synthesized Mg-Ni hollow nanospheres, where the hollow structure of Mg enhanced hydrogen storage performance, achieving a storage density of 4.9 wt%, compared to 3.8 wt% for non-hollow nanospheres [21]. Similar results have been observed in Li-based materials [22]. David et al. demonstrated that hollow structures not only reduce adsorption temperatures but also increase adsorption rates. Li_2_NH hollow nanospheres adsorbed 6 wt% hydrogen at 473 K in 60 s, whereas Li_2_NH micrometer particles adsorbed only 1.6 wt% under the same conditions [23]. Thus, internal hollow structures can effectively enhance hydrogen storage density. Recent advances in synthesizing materials with hydrogen storage potential have significantly propelled hydrogen storage technology. Hollow spheres, characterized by low density and high surface area, are among the most promising alternative materials for hydrogen storage [24].

During chemical adsorption, hydrogen exists in atomic form, making dissociation challenging. However, in physical adsorption on materials with high surface areas, the binding force between hydrogen molecules and the substrate is weaker, requiring lower energy for desorption, which can occur at lower temperatures [25]. Therefore, to achieve reversible hydrogen storage at ambient temperature and pressure, the binding energy between the substrate and hydrogen should lie between physical and chemical adsorption [26]. Physical adsorption, also known as van der Waals adsorption, typically requires low temperatures to achieve reasonable storage capacities. In contrast, chemical hydrogen storage requires relatively high operating temperatures due to the breaking of strong chemical bonds during dehydrogenation. A promising hydrogen storage system should exhibit good reversibility at ambient temperatures, with adsorption capacities greater than 0.25 eV for chemical adsorption and less than 0.25 eV for physical adsorption [25]. Thus, the ideal binding strength between hydrogen molecules and the substrate should range from 0.1 eV to 0.8 eV [27,28,29]. To date, no system has achieved satisfactory performance levels due to interactions that are either too weak (physical adsorption) or too strong (chemical adsorption). Extensive research is needed to improve known materials or invent more efficient systems. Recent developments in nanomaterials and cluster science have provided new insights into hydrogen storage technology. Cluster materials, with their unique geometric structures, high surface areas, and tunable physicochemical properties, show great potential in hydrogen storage. Particularly, hollow cage clusters, with their internal cavities and surface active sites, can effectively adsorb and store hydrogen. Among the second-period elements, those with low atomic masses are ideal candidates. Carbon and boron-based materials exhibit weak interactions with hydrogen molecules, resulting in low storage densities [30,31,32]. Lithium, a first-group element, forms strong ionic bonds with hydrogen, making hydrogen release difficult and resulting in high adsorption energies [33]. Boron, a third-group element, typically forms covalent bonds or complex hydrides with hydrogen, exhibiting low stability but high reactivity, with low adsorption energies [34,35]. Beryllium, a second-group element, offers an intermediate interaction strength between lithium and boron, making Be-based hollow spherical clusters a hot research topic due to their low atomic mass, high stability, and excellent hydrogen storage performance [25,36,37]. This study aims to investigate the structural properties of Be_n_(n = 10–12) hollow spherical clusters and their potential applications in hydrogen storage. Utilizing theoretical calculations, we analyze the geometric configurations of Be_n_(n = 10–12) hollow spherical clusters, the adsorption behavior of hydrogen molecules on their surfaces, as well as the electronic properties and desorption temperatures following hydrogen adsorption. It should be noted that beryllium and its compounds are toxic, which must be carefully considered in any potential experimental synthesis or handling of Be-based materials. Furthermore, we evaluate their hydrogen storage capacity and kinetic performance. The results of this research are expected to provide a theoretical foundation and technical support for the design of novel, high-efficiency hydrogen storage materials, thereby contributing to the advancement of hydrogen energy technology.

## 2. Results and Discussion

### 2.1. Stable Structures

**Figure 1:**
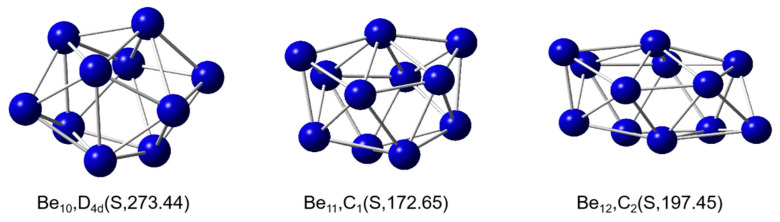
The lowest-energy structures of Be_n_(n = 10–12) clusters with exact symmetries, point group symmetry and state (S = singlet), the minimum vibrational frequency (cm^−1^) in parentheses.

To investigate the hydrogen storage performance of Be_n_(n = 10–12) clusters, we optimized the structures of numerous Be_n_(n = 10–12) cluster isomers using Gaussian 16 software and determined their stability through frequency analysis. The lowest-energy structures of Be_n_(n = 10–12) cluster complexes are shown in Figure 1. As depicted, Be_n_(n = 10–12) clusters are all hollow spherical clusters. Be_10_ is a singlet with D_4_d point group symmetry, with Be-Be bond lengths ranging from 2.039 Å to 2.203 Å and an average bond length of 2.104 Å. Be_11_ is a singlet with C1 point group symmetry, with Be-Be bond lengths ranging from 2.003 Å to 2.260 Å and an average bond length of 2.114 Å. Be_12_ is a singlet with C2 point group symmetry, with Be-Be bond lengths ranging from 1.984 Å to 2.324 Å and an average bond length of 2.112 Å. In the study by Abyaz et al., the ground state of Be_11_ was reported as a triplet. However, in our work, based on calculations performed at the PBE0-D3/DEF2-TZVP theoretical level, we found that the lowest-energy structure of Be_11_ is a singlet (with C_1_ symmetry). Abyaz et al. may have employed a different functional or basis set that did not include dispersion corrections. In contrast, in our study, we explicitly incorporated D3 dispersion corrections into the PBE0 functional, which significantly influences the energy ordering of weakly interacting systems, such as beryllium clusters. Other computational results are consistent with those reported in the study by Abyaz et al. [38].

We conducted a basis set test using the higher-precision computational method-developed domain-based local pair natural orbital coupled-cluster theory with TightPNO truncation threshold (DLPNO-CCSD(T)). The results are shown in Table 1. It was found that the energy difference between the PBE0-D3 method and the DLPNO-CCSD(T) method is not significant, indicating that the use of the PBE0-D3 method is feasible.

### 2.2. Hydrogen Storage Performance

**Figure 2:**
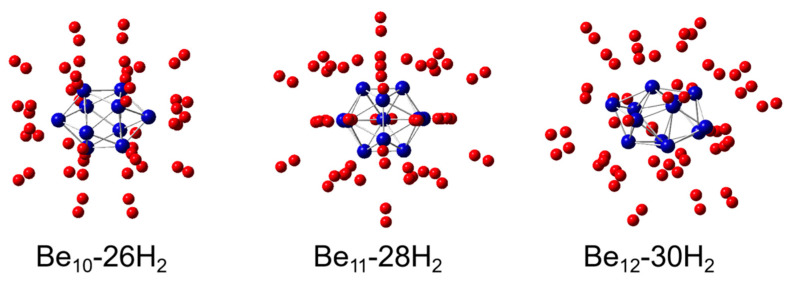
The most stable state of Be_n_(n = 10–12) adsorbed multiple H_2_ molecules obtained after geometric optimization.

The lowest-energy structures of Be_n_(n = 10–12)@H_2_ complexes are shown in Figure 2. The figure clearly illustrates the adsorption of hydrogen molecules on Be_n_(n = 10–12) clusters. In all three structures, we observed that H_2_ molecules initially adsorb onto Be atomic sites, followed by adsorption onto the face sites formed by three Be atoms. After the Be_10_ cluster adsorbs 26 H_2_ molecules, the Be_11_ cluster adsorbs 28 H_2_ molecules, and the Be_12_ cluster adsorbs 30 H_2_ molecules, no new vacancies are available for further adsorption of H_2_ molecules. The relevant data have been uploaded to the Supplementary Materials. All three clusters are hollow spherical structures, which aligns with the findings of Majid Zarezadeh Mehrizi and Jafar Abdi [24], who suggested that hollow spherical clusters exhibit superior hydrogen adsorption due to their lower volumetric density and higher surface area. In hollow spherical clusters, the number of adsorption sites increases with the same number of Be atoms, and the volumetric density decreases. Given that Be has a low atomic mass (9.012), these clusters achieve exceptionally high hydrogen storage densities.

**Figure 3:**
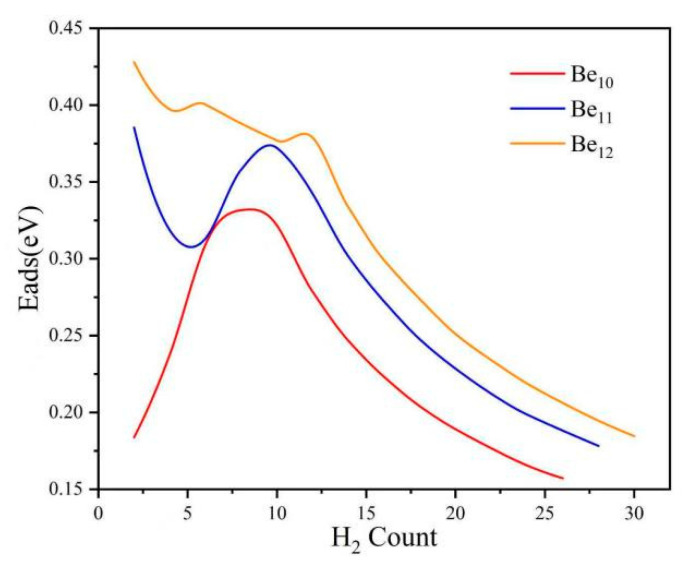
Stepwise hydrogen adsorption energies.

All clusters exhibit average adsorption energies within the range of 0.16 eV to 0.19 eV/H_2_ is shown in Table 2. Figure 3 shows that the adsorption energies of all clusters are in the range of 0.15 eV to 0.43 eV, all within the ideal binding strength range of 0.1–0.8 eV for hydrogen and the substrate. Thus, we consider that saturated adsorption has been reached. The positive Eads values indicate that the H_2_ adsorption process is exothermic [39]. These Eads values fall within the range of physical adsorption, suggesting that the considered clusters are suitable for reversible H_2_ adsorption at room temperature and pressure. Under 0 K conditions, Be_n_(n = 10–12) clusters can bind up to 30 H_2_ molecules. As shown in Table 1, it was found that the Be_12_-30H_2_ cluster achieves the highest hydrogen storage density (35.87 wt%), followed by Be_10_-26H_2_ (31.96 wt%) and Be_11_-28H_2_ (31.87 wt%). All Be_n_(n = 10–12) clusters exceed the international standard of 5.5 wt%, showing promise as a novel class of nanomaterials for hydrogen storage.

### 2.3. Electronic Properties

To gain deeper insights into the bonding characteristics of the clusters and their corresponding H_2_-bonded complexes, we conducted partial density of states (PDOS) calculations. PDOS is a further decomposition of the density of states (DOS), used to analyze the contributions of specific atomic orbitals or atoms to the total density of states. PDOS analysis is a crucial tool for studying the electronic structures of materials, helping to understand the roles of different atoms or orbitals in the band structure.

**Figure 4:**
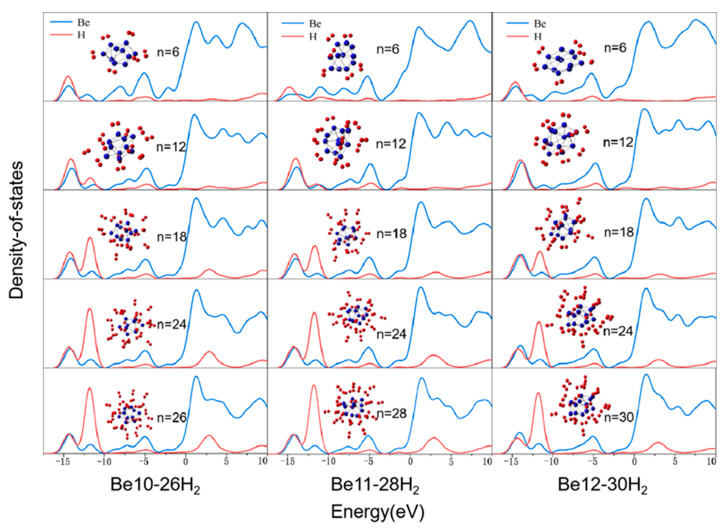
The split-wave projected density of states (E_f_ = 0 eV) where n represents the number of hydrogen molecules adsorbed on the clusters.

Therefore, we conducted Projected Density of States (PDOS) calculations to investigate the interactions between the atomic orbitals within the system. We calculated the PDOS for the 1s orbitals of hydrogen atoms and the 2s orbitals of H and Be atoms, as shown in Figure 4. The figure clearly shows significant overlap between the DOS curves of H and Be atoms at −14.5 eV, indicating strong orbital hybridization interactions. Similarly, there is a slight overlap between the 1s orbitals of H atoms and the 2s orbitals of Be atoms, suggesting weak orbital hybridization interactions. As the number of hydrogen molecules increases, the PDOS curves exhibit peak growth and splitting, indicating increased electron density and repulsion between H molecules, leading to different energy levels. With increasing hydrogen molecule numbers, the electron density of H molecules between −15 eV and −10 eV gradually increases, while the peaks of Be remain largely unchanged, indicating stable electronic density distribution in Be_n_(n = 10–12) clusters after hydrogen adsorption. The peak widths in the PDOS curves do not change significantly with increasing hydrogen molecule numbers, indicating consistent chemical bond or interaction strengths within the analyzed energy range, high structural stability, and uniform electronic state distribution. The peak widths around 5 eV correspond to delocalized electronic states, indicating weak intermolecular interactions dominated by van der Waals forces, consistent with physical adsorption characteristics.

**Figure 5:**
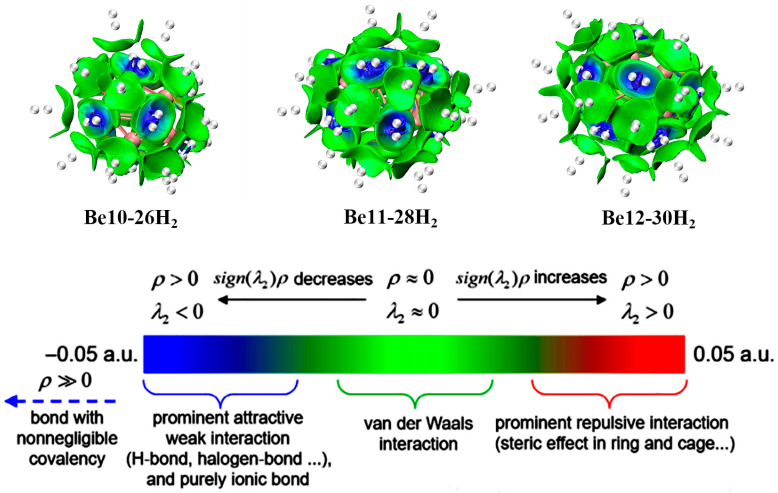
$sign\left(\lambda_{2}\right)\rho$ Isosurface color corresponds to IGMH analysis between H_2_ and Be_n_(n = 10–12). Mapping function symbols $sign\left(\lambda_{2}\right)\rho$ in IGMH Mapping, a general explanation of the coloring method.

Figure 5 illustrates the color coding in the IGMH isosurface, where green represents van der Waals forces, blue indicates weak attractive interactions (typically hydrogen bonds), and red signifies repulsive forces. Figure 4 presents the results obtained from our IGMH (Independent Gradient Model based on Hirshfeld partitioning) calculations. By comparing it with Figure 4, we can elucidate the nature of the interactions between H_2_ and the Be_n_(n = 10–12) clusters.

The independent gradient model based on Hirshfeld partitioning (IGMH) defines actual electron density, providing a more rigorous physical interpretation and accurately reflecting the electronic structure of the studied system. Notably, Hirshfeld partitioning is a common method for obtaining atomic densities in chemical systems, offering clear physical images [40,41,42]. In this section, we focus on using the IGMH method to reveal weak interactions in the Be_n_ system. We plotted the colored isosurface map, as shown in Figure 5. In the calculations, each H_2_ molecule was defined as a separate fragment, so Figure 5 only shows the interactions between H_2_ and Be_n_(n = 10–12). The IGMH map reveals thin and wide isosurfaces between each H_2_ and Be_n_(n = 10–12), ideally representing π-π stacking interactions. The isosurface colors indicate that H_2_ molecules on the cluster surfaces are light blue and green, suggesting that H_2_ adsorption on Be_n_(n = 10–12) is predominantly influenced by van der Waals forces, consistent with physical adsorption characteristics.

### 2.4. Desorption Temperature

In hydrogen storage material research, desorption temperature is a critical parameter reflecting the ease of hydrogen release from the storage medium. Analyzing desorption temperatures is essential for evaluating the practical application performance of hydrogen storage materials. We calculated the desorption temperatures at 1 atm, 2 atm, 3 atm, 5 atm, and 10 atm. Materials with lower desorption temperatures typically exhibit higher hydrogen storage capacities, as hydrogen is more easily released. They also demonstrate better cycling stability due to higher reversibility in hydrogen release and adsorption. Such materials are more suitable for practical applications, as hydrogen can be released at lower temperatures, reducing energy consumption [43].

**Figure 6:**
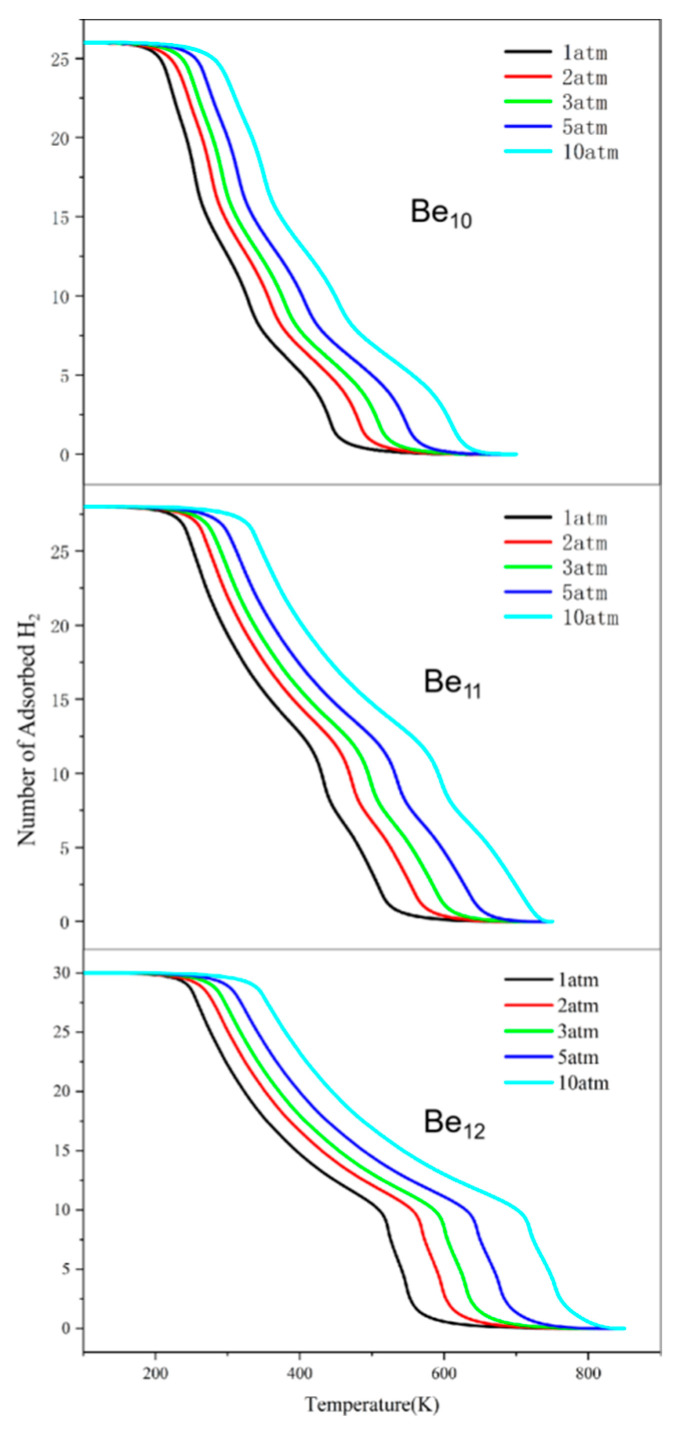
The thermodynamics of H_2_ molecule adsorption on the substrate at different temperatures (T) and pressures (p).

Our results reveal a desorption trend, as illustrated in Figure 6. With increasing temperature, we observe a gradual reduction in the number of hydrogen molecules that can be adsorbed on the Be_n_(n = 10–12) clusters. At a pressure of 1 atm, the desorption of H_2_ molecules from the Be_10_-26H_2_ cluster begins at 216 K, from the Be_11_-28H_2_ cluster at 245 K, and from the Be_12_-30H_2_ cluster at 254 K. As the pressure increases, the desorption temperature of the hydrogen molecules also exhibits an upward trend. These findings collectively indicate that the Be_n_(n = 10–12) hollow spherical clusters can be regarded as materials capable of reversible hydrogen storage at temperatures above 216 K. As shown in Figure 6, increasing the pressure to 5–10 atm raises the desorption temperature above 298 K, allowing reversible hydrogen storage and release under near-ambient conditions. Furthermore, by increasing the pressure, reversible hydrogen storage remains feasible at elevated temperatures.

## 3. Methods

In this study, we employed density functional theory (DFT) to investigate the electronic properties and hydrogen storage performance of Be_n_(n = 10–12) clusters. All calculations were performed using Gaussian 16 software [44]. We initially optimized the structures of numerous Be_n_(n = 10–12) cluster isomers and determined their stability through frequency analysis. Subsequently, we applied a screening method to identify the lowest energy structures of Be_n_(n = 10–12) clusters. All calculations were conducted at the PBE0-D3/DEF2-TZVP [45,46] theoretical level using Gaussian 16 software. The 1996 pure functional of Perdew, Burke, and Ernzerhof was hybridized by Adamo, utilizing 25% exchange and 75% correlation weights [47]. To account for weak interactions (van der Waals forces, hydrogen bonds) between hydrogen molecules and beryllium clusters, we incorporated D3 dispersion correction into PBE0-D3/DEF2-TZVP, effectively handling long-range van der Waals interactions. This is crucial for weak interaction systems, such as intermolecular interactions and adsorption. The D3 correction significantly enhances the prediction accuracy of intermolecular interaction energies. The DEF2-TZVP basis set, which includes more atomic orbitals and polynomial basis functions, provides more accurate molecular structure descriptions and energy calculations. DEF2-TZVP offers high precision, suitable for describing electronic structures of main-group and transition metal elements, and is efficient for medium-sized systems [46]. Thus, using PBE0-D3/DEF2-TZVP as the basis set for Be_n_(n = 10–12) cluster calculations is appropriate. After obtaining the stable structures of Be_n_(n = 10–12) clusters, we sequentially adsorbed two H_2_ molecules onto these structures. The average adsorption energy (*Eads*) was calculated using the following equation:

(1) $$E_{ads}=\left[E\left(cluster\right)-nE\left(H_{2}\right)-E\left(complex\right)\right]/n$$

where E(cluster), $E\left(H_{2}\right)$ and E(complex) are the energies of the Be_n_(n = 10–12) cluster, H_2_ molecule, and H_2_-bonded cluster, respectively. The hydrogen storage density of Be_n_(n = 10–12) clusters was calculated using the following equation:

(2) $$\omega t\%=\left[\frac{M_{H_{2}}}{M_{H_{2}}+M_{cluster}}\right]$$

where $M_{H_{2}}$ and $M_{cluster}$ are the masses of H_2_ and the Be_n_(n = 10–12) clusters, respectively.

To gain deeper insights into the bonding characteristics of the clusters and their corresponding H_2_-bonded complexes, we performed partial density of states (PDOS) calculations using Multiwfn3.0 software. Multiwfn is an open-source software for molecular and solid-state electronic structure analysis [48]. It offers a wide range of functionalities, including molecular orbital analysis, electron localization indicator calculations, charge distribution analysis, atomic shell analysis, and intermolecular interaction analysis. Multiwfn supports various input file formats, including outputs from Gaussian, CP2K, and other quantum chemistry and first-principle calculation programs. The software features a user-friendly graphical interface and supports command-line operations to meet diverse user needs. Due to its rich functionality, ease of use, and open-source nature, Multiwfn is widely used in chemistry, materials science, and biological science research.

The density of states *g*(*E*) is defined as the number of electronic states per unit energy interval, expressed mathematically as:

(3) $$g(E)=\sum_{i}\sigma (E-E_{i})$$

where $E_{i}$ is the energy of the *i*th electronic state, and $\sigma (E-E_{i})$ is the Dirac σ delta function, indicating that $E_{i}$ has one state. In practical calculations, the Dirac delta function is often approximated by Gaussian or Lorentzian functions for numerical computation:

(4) $$g(E)\approx \sum_{i}\frac{1}{\sigma \sqrt{2\pi }}\exp\left(-\frac{(E-E_{i}{)}^{2}}{2\sigma^{2}}\right)$$

where σ is the broadening parameter controlling the peak width. The partial density of states (PDOS) represents the contribution of specific atoms or orbitals to the total density of states. Multiwfn also supports PDOS calculations, with the formula:

(5) $$g_{PDOS}(E)=\sum_{i\in \mathrm{Specific}\;\mathrm{tracks}}\sigma (E-E_{i})$$

where Dirac σ can also be approximated by Gaussian or Lorentzian functions.

To explore the intramolecular interactions within Be_n_ (n = 10–12) clusters, we employed the IGMH isosurface method [49]. The IGMH isosurface method is a chemical bond analysis tool based on electron density and its Laplacian, providing intuitive visualization of bond strengths and distributions. Multiwfn offers convenient IGMH calculation and visualization functionalities, suitable for research in chemistry and materials science. By appropriately setting isosurface parameters, one can deeply analyze intramolecular and intermolecular interactions. The IGMH index quantifies bond strength by combining electron density and its Laplacian, with the formula:

(6) $$IGMH(r)=\frac{\left|\nabla \rho (r)\right|}{\rho (r)}$$

where ρ(r) describes the electron distribution in space. A higher IGMH index indicates stronger bonds. By setting IGMH isosurfaces, one can visualize bond strengths and distributions in three-dimensional space, with isosurface values adjustable based on specific research needs.

Desorption temperature (TD) is a key parameter for investigating the feasibility of hydrogen storage materials, which reflects the temperature required for hydrogen molecules to be released from the storage medium. In this study, the van’t Hoff equation was used to calculate the desorption temperature of hydrogen molecules on the substrate:

(7) $$T_{D}=\frac{E_{ads}}{k_{B}}{\left(\frac{∆S}{R}-\ln p\right)}^{-1}$$

where *k_B_* is Boltzmann constant (1.38 × 10^−23^ J/K), R is the gas constant (8.31 J/K), p is the equilibrium pressure, ∆S represents the entropy change of hydrogen from gaseous to liquid state (70 J/mol⋅K), and Eads is the average adsorption energy of hydrogen molecules on the substrate [50].

## 4. Conclusions

Based on density functional theory (DFT), we conducted an in-depth investigation into the structural stability, hydrogen storage performance, electronic properties, and desorption temperature of Be_n_(n = 10–12) hollow spherical clusters after hydrogen adsorption. The results demonstrate that the Be_n_(n = 10–12) hollow spherical clusters exhibit excellent hydrogen storage performance, and their structures remain largely unchanged after hydrogen adsorption. The average adsorption energy of the Be_n_(n = 10–12) hollow spherical clusters falls within the range of 0.16 eV to 0.19 eV/H_2_. The stepwise adsorption energies range from 0.15 eV to 0.43 eV, indicating that the hydrogen storage mechanism is governed by van der Waals forces and chemical bonding interactions between the H_2_ molecules and the substrate material. Under 0 K conditions, the Be_n_(n = 10–12) hollow spherical clusters can adsorb 26, 28, and 30 hydrogen molecules, respectively. The theoretical maximum hydrogen storage densities for these clusters at 0 K all exceed 31 wt%, reaching up to 35.87 wt%, with the highest reaching 35.87 wt%, significantly surpassing the U.S. Department of Energy’s target of 5.5 wt%. Analysis of the projected density of states (PDOS) and IGMH isosurfaces for hydrogen molecules adsorbed on the substrate reveals characteristics consistent with physical adsorption. Notably, our computational results indicate that H_2_ molecules can desorb at temperatures above 216 K. Increasing the pressure to 5–10 atm raises the desorption temperature above 298 K, allowing reversible hydrogen storage and release under near-ambient conditions, further validating the potential of this material as a reversible hydrogen storage medium. By virtue of their high specific surface area, the hollow spherical Be_n_(n = 10–12) clusters exhibit significantly enhanced hydrogen storage performance, which renders them a promising candidate for novel hydrogen storage nanomaterials.

## Figures and Tables

**Table 1:** The energy difference between the PBE0-D3 and DLPNO-CCSD(T) methods is negligible.

Cluster	Method	Energy (ev)
Be_10_	DLPNO-CCSD(T) PBE0-D3	4003.51 4000.79
Be_11_	DLPNO-CCSD(T) PBE0-D3	4404.37 4401.29
Be_12_	DLPNO-CCSD(T) PBE0-D3	4804.21 4801.44

**Table 2:** Average adsorption energy and hydrogen storage density of Be_n_(n = 10–12) clusters are calculated.

Cluster	Eads (eV)	wt%
Be_10_-26H_2_	0.16	31.96
Be_11_-28H_2_	0.18	31.87
Be_12_-30H_2_	0.19	35.87

## Data Availability

The original contributions presented in this study are included in the article and Supplementary Materials. Further inquiries can be directed to the corresponding author.

## Supplementary Materials

The following supporting information can be downloaded at: https://www.mdpi.com/article/10.3390/molecules31030566/s1. The Supplementary Material provides the raw data detailing the calculated structure and energy of H_2_, the stable structures and energies of Be_n_(n = 10–12), and the stable structures and energies of Be_n_(n = 10–12) during the hydrogen adsorption process.
